# Comparison of ^68^Ga-DOTA-Siglec-9 and ^18^F-Fluorodeoxyribose-Siglec-9: Inflammation Imaging and Radiation Dosimetry

**DOI:** 10.1155/2017/7645070

**Published:** 2017-12-31

**Authors:** Helena Virtanen, Johanna M. U. Silvola, Anu Autio, Xiang-Guo Li, Heidi Liljenbäck, Sanna Hellberg, Riikka Siitonen, Mia Ståhle, Meeri Käkelä, Anu J. Airaksinen, Kerttuli Helariutta, Tuula Tolvanen, Tibor Z. Veres, Antti Saraste, Juhani Knuuti, Sirpa Jalkanen, Anne Roivainen

**Affiliations:** ^1^Turku PET Centre, University of Turku, Turku, Finland; ^2^Turku PET Centre, Åbo Akademi University, Turku, Finland; ^3^Laboratory of Radiochemistry, Department of Chemistry, University of Helsinki, Helsinki, Finland; ^4^Turku Centre for Disease Modeling, University of Turku, Turku, Finland; ^5^Turku PET Centre, Turku University Hospital, Turku, Finland; ^6^MediCity Research Laboratory, University of Turku, Turku, Finland

## Abstract

Sialic acid-binding immunoglobulin-like lectin 9 (Siglec-9) is a ligand of inflammation-inducible vascular adhesion protein-1 (VAP-1). We compared ^68^Ga-DOTA- and ^18^F-fluorodeoxyribose- (FDR-) labeled Siglec-9 motif peptides for PET imaging of inflammation.* Methods*. Firstly, we examined ^68^Ga-DOTA-Siglec-9 and ^18^F-FDR-Siglec-9 in rats with skin/muscle inflammation. We then studied ^18^F-FDR-Siglec-9 for the detection of inflamed atherosclerotic plaques in mice and compared it with previous ^68^Ga-DOTA-Siglec-9 results. Lastly, we estimated human radiation dosimetry from the rat data.* Results*. In rats, ^68^Ga-DOTA-Siglec-9 (SUV, 0.88 ± 0.087) and ^18^F-FDR-Siglec-9 (SUV, 0.77 ± 0.22) showed comparable (*P* = 0.29) imaging of inflammation. In atherosclerotic mice, ^18^F-FDR-Siglec-9 detected inflamed plaques with a target-to-background ratio (1.6 ± 0.078) similar to previously tested ^68^Ga-DOTA-Siglec-9 (*P* = 0.35). Human effective dose estimates for ^68^Ga-DOTA-Siglec-9 and ^18^F-FDR-Siglec-9 were 0.024 and 0.022 mSv/MBq, respectively.* Conclusion*. Both tracers are suitable for PET imaging of inflammation. The easier production and lower cost of ^68^Ga-DOTA-Siglec-9 present advantages over ^18^F-FDR-Siglec-9, indicating it as a primary choice for clinical studies.

## 1. Introduction

Inflammation plays role in several diseases, such as, rheumatoid arthritis, diabetes and atherosclerosis. The early detection of inflammatory foci is critical for the adequate treatment of patients, and quantitative PET imaging may provide a valuable tool for diagnosis and monitoring of the effects of therapeutic interventions. ^18^F-FDG is the gold standard for PET, but not specific to inflammation. In addition, the high physiological accumulation of ^18^F-FDG in heart and brain makes it difficult to detect inflammatory foci close to these organs [1].

Vascular adhesion protein-1 (VAP-1) is an endothelial adhesion molecule, which is involved in leukocyte transendothelial migration from blood into the sites of inflammation. During inflammation VAP-1 translocates from intracellular storages on the endothelial cell surface where it contributes leukocyte-endothelial adhesion. Although VAP-1 plays important role in early phases of inflammation, its luminal expression on the endothelium will remain constant if the inflammation continues, which suggest VAP-1 as a promising target for both anti-inflammatory therapy and molecular imaging of inflammation [2]. The role of VAP-1 in atherosclerotic inflammation is unclear. VAP-1 expression is upregulated in atherosclerotic plaques in human carotid arteries [3, 4] and in the aorta of hypercholesterolemic rabbits [5]. VAP-1 is also expressed in soluble form (sVAP-1), which associates with atherosclerosis [6, 7].

We previously showed that sialic acid-binding immunoglobulin-like lectin 9 (Siglec-9) is a VAP-1 ligand, and the radiolabeled peptide (CARLSLSWRGLTLCPSK) containing residues 283–297 from Siglec-9 can be used for PET imaging of inflammation [4, 8–11].

In this study, we examined the detection of skin/muscle inflammation in rats, comparing the utility of the Siglec-9 motif peptides ^68^Ga-labeled 1,4,7,10-tetraazacyclododecane-N,N′,N′′,N′′′-tetraacetic acid-conjugated ^68^Ga-DOTA-Siglec-9 and ^18^F-labeled fluorodeoxyribose-conjugated ^18^F-FDR-Siglec-9 [9]. We also examined ^18^F-FDR-Siglec-9 uptake in inflamed atherosclerotic plaques of mice and compared it with previous ^68^Ga-DOTA-Siglec-9 results [4]. Finally, we used the rat PET data to estimate the human radiation doses for ^68^Ga-DOTA-Siglec-9 and ^18^F-FDR-Siglec-9.

## 2. Materials and Methods

### 2.1. Radiochemistry

^68^Ga-DOTA-Siglec-9 and ^18^F-FDR-Siglec-9 were produced as previously described [8, 9].

### 2.2. Animal Models

Twenty-four hours before the PET studies, Sprague-Dawley rats (weight, 350 ± 22 g; *n* = 16) were subcutaneously injected with turpentine oil (Sigma-Aldrich) to induce focal acute, sterile inflammation [12]. Before the injection, rats were shaved on the both forelegs. Inflamed area on the left foreleg contained both skin and muscle. The intact, contralateral side (right foreleg) was used as a control.

Six-month-old atherosclerotic low-density lipoprotein receptor-deficient mice (weight, 27 ± 4.1 g; *n* = 19) expressing only apolipoprotein B100 (LDLR^−/−^ApoB^100/100^, strain #003000; Jackson Laboratory, Bar Harbor, ME, USA) were fed for 4 months with a Western-type diet [13]. Two-month-old C57BL/6N mice (weight, 24 ± 2.2 g; *n* = 13) fed with a regular chow served as controls.

All animal experiments (Table 1) were approved by the national Animal Experiment Board in Finland and carried out in compliance with the EU directive.

### 2.3. Rat Studies

Rats were divided into two groups with Group 1 being intravenously (i.v.) given ^68^Ga-DOTA-Siglec-9 (16 ± 2.9 MBq, *n* = 8) and Group 2 ^18^F-FDR-Siglec-9 (18 ± 5.1 MBq, *n* = 8). A 60 min dynamic PET acquisition was performed on a High Resolution Research Tomograph (HRRT; Siemens Medical Solutions, Knoxville, TN, USA). The PET data were reconstructed into 5 × 60 s and 11 × 300 s frames using an ordered-subsets expectation maximization 3D algorithm (OSEM3D). Quantitative PET image analysis was performed by defining regions of interest (ROIs) within the inflamed area (on the left foreleg), control area (on the right foreleg), kidneys, lungs, heart, liver, and urinary bladder using Carimas 2.8 software (Turku PET Centre). Results were expressed as standardized uptake values (SUV) and time-activity curves. SUV was calculated as a ratio of tissue radioactivity concentration (Bq/mL) and given radioactivity dose (Bq) divided by animal's body weight.

After PET imaging, rats were sacrificed and various tissues were excised and weighed, and their radioactivity levels were measured with a gamma counter (1480 Wizard 3′′, PerkinElmer, Turku, Finland). The* ex vivo* biodistribution results were expressed as a percentage of the injected radioactivity dose per gram of tissue (% ID/g) and target-to-background ratio.

The inflamed area and control area tissue samples were frozen, cut into sections, and stained with hematoxylin-eosin (H&E) for morphological evaluation.

Absorbed doses of ^68^Ga-DOTA-Siglec-9 and ^18^F-FDR-Siglec-9 were calculated with the OLINDA/EXM version 1.0 software (organ level internal dose assessment and exponential modeling; Vanderbilt University, Nashville, TN, USA), which applies the MIRD schema (developed by the Medical Internal Radiation Dose committee of the Society of Nuclear Medicine) for radiation dose calculations in internal exposure. The software includes radionuclide information and selection of human body phantoms. The residence times derived from the rat data were integrated as the area under the time-activity curve. The residence times were converted into corresponding human values by multiplication with a factor to scale the organ and body weights: (W_Body,rat_/W_Organ,rat_)×(W_Organ,human_/W_Body,human_), where W_Body,rat_ and W_Body,human_ are the body weights of rat and human (a 70-kg male), respectively; and *W*_Organ,rat_ and *W*_Organ,human_ are the organ weights of rat and human (organ weights for a 70 kg male), respectively [14].

### 2.4. Mouse Studies

To detect luminal expression of VAP-1, mice were intravenously (i.v.) injected with a monoclonal rat anti-mouse VAP-1 antibody (7–88, 1 mg/kg diluted in saline) [15] 10 min before sacrifice. Aorta samples were frozen and cut into 8 *μ*m longitudinal sections, incubated for 30 min at room temperature in the dark with a secondary goat anti-rat antibody (working dilution, 5 *μ*g/mL in phosphate-buffered saline (PBS) containing 5% normal mouse or human AB serum), conjugated to a fluorescent dye (Alexa Fluor 488; Invitrogen, Eugene, OR, USA), and rinsed twice in PBS for 5 min.

In PET studies, mice (19 atherosclerotic, 13 controls) were injected with 14 ± 4.4 MBq of ^18^F-FDR-Siglec-9. Twenty-five minutes after ^18^F-FDR-Siglec-9 injection, blood was drawn by cardiac puncture and the animals were killed. The thoracic aorta was excised and rinsed in saline to remove the blood. In addition, various other tissues were excised and patted dry. Samples of blood and urine were collected, and blood plasma was separated by centrifugation. All tissue samples were weighed, and their radioactivity levels were measured with a gamma counter (Triathler 3′′, Hidex Oy, Turku, Finland). The results were expressed as % ID/g and target-to-background ratio.

Autoradiography was used to study the distribution of radioactivity in the aorta in more detail, as described previously [13]. After careful superimposition of the autoradiographs and H&E stained images, the count densities of 540 ROIs (185: plaques, 241: normal vessel walls, and 114: adventitia) were analyzed using Tina 2.1 software. The autoradiography results were calculated as the photostimulated luminescence per unit area (PSL/mm^2^) normalized for injected radioactivity dose, and as ratios between the atherosclerotic plaque, normal vessel wall, and adventitia.

A subset of mice (two atherosclerotic, two controls) were injected with 4.7 ± 1.1 MBq of ^18^F-FDR-Siglec-9 and imaged with an Inveon Multimodality PET/CT (Siemens, Medical Solutions, Knoxville, TN, USA). Dynamic PET images were acquired for 60 min, followed by CT with a contrast agent (eXIATM160XL, Binitio Biomedical Inc., Ottawa, ON, Canada). The PET images were reconstructed with OSEM3D (frames 5 × 60 s, 3 × 300 s, 1 × 600 s, 2 × 1800 s).

Quantitative PET image analysis was performed by defining ROIs in the heart left ventricle (blood pool) and aortic arch as identified on the basis of the CT angiography by using the Inveon Research Workplace software (Siemens Medical Solutions, Knoxville, TN, USA). Time frames 10–20 min after injection were used for PET quantification, as previously reported in the same mouse model using ^68^Ga-DOTA-Siglec-9 [4]. The results within ROIs were expressed as SUV and target-to-background ratio (SUV_max,aortic  arch_/SUV_mean,blood_).

Finally, we compared ^18^F-FDR-Siglec-9 results with previously reported ^68^Ga-DOTA-Siglec-9 [4].

### 2.5. Statistical Analyses

All results are expressed as mean ± SD. Paired 2-tailed Student's *t*-tests were applied for intra-animal comparisons. Nonpaired data were compared between two groups using *t*-tests and between multiple groups using ANOVA with Tukey's correction. A *P* value less than 0.05 was considered statistically significant.

## 3. Results

### 3.1. Radiochemistry

The specific radioactivity of ^68^Ga-DOTA-Siglec-9 and ^18^F-FDR-Siglec-9 was 70 ± 15 MBq/nmol and 83 ± 33 MBq/nmol, respectively, with radiochemical purity being >95% throughout the study.

### 3.2. Rat Studies

**Figure 1:**
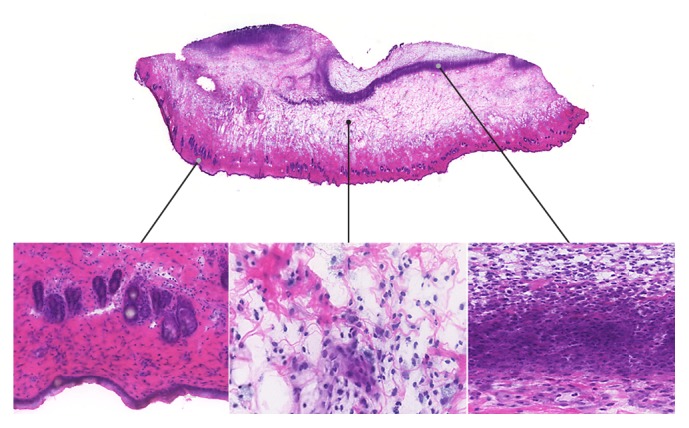
Inflammation of skin/muscle in a rat at 24 hours after subcutaneous injection of turpentine oil. Hematoxylin-eosin staining of a 10 *μ*m cryosection reveals edema and leukocyte infiltration, predominantly neutrophils.

The turpentine oil caused focal soft-tissue inflammation with edema and leukocyte infiltration, predominantly neutrophils (Figure 1), and luminal VAP-1 as previously reported [8, 16].

**Figure 2:**
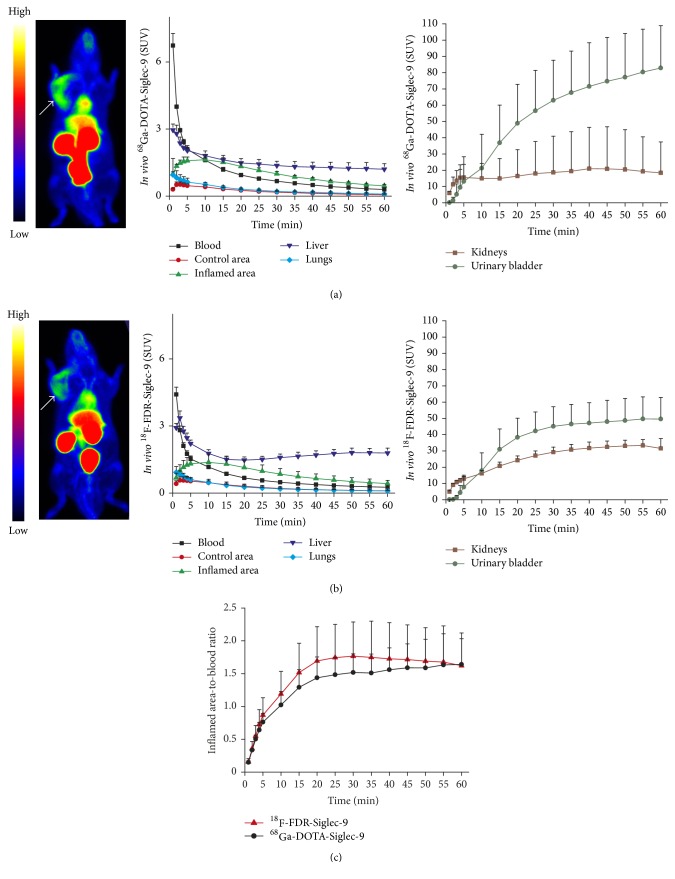
PET images and time-activity curves of rats with skin/muscle inflammation (arrow) imaged with (a) ^68^Ga-DOTA-Siglec-9 (*n* = 5) or (b) ^18^F-FDR-Siglec-9 (*n* = 8) and (c) comparison of their target-to-blood kinetics.

The inflammatory focus was clearly visualized with both of the PET tracers and was demonstrated in the time-activity curves of the inflamed area, with the uptake kinetics of both tracers being comparable (Figure 2). In the inflamed area, the SUV_mean,10–60 min_ of ^68^Ga-DOTA-Siglec-9 and ^18^F-FDR-Siglec-9 was 0.88 ± 0.087 and 0.77 ± 0.22, respectively (*P* = 0.29). The corresponding SUV_max,10–60 min_ values for ^68^Ga-DOTA-Siglec-9 (1.1 ± 0.10) and ^18^F-FDR-Siglec-9 (1.1 ± 0.097) were also close to each other (*P* = 0.47). Both tracers peaked about 10 min after the i.v. bolus injection, with a slow decrease thereafter. The inflammation-to-blood ratios_10–60 min_ of ^68^Ga-DOTA-Siglec-9 (1.5 ± 0.33) and ^18^F-FDR-Siglec-9 (1.7 ± 0.51) were also comparable (*P* = 0.54). Uptake in the heart, liver, kidneys, and urinary bladder was clearly visible with both tracers (Figure 2).

*Ex vivo* gamma counting at 60 min after injection demonstrated that ^18^F-FDR-Siglec-9 uptake was significantly higher than ^68^Ga-DOTA-Siglec-9 uptake in several organs, including inflamed and control areas. The difference was particularly notable in the liver, pancreas, heart, and kidneys (Table 2). Only in the spleen was the uptake of ^68^Ga-DOTA-Siglec-9 significantly higher than that of ^18^F-FDR-Siglec-9. Although the uptake of ^18^F-FDR-Siglec-9 in inflamed area (0.19 ± 0.053  % ID/g) was significantly higher (*P* = 0.013) than that of ^68^Ga-DOTA-Siglec-9 (0.12 ± 0.032  % ID/g), the inflammation-to-blood and inflamed-to-control area ratios (^18^F-FDR-Siglec-9: 1.3 ± 0.16 and 2.0 ± 0.70; ^68^Ga-DOTA-Siglec-9: 1.4 ± 0.42 and 2.5 ± 0.54) were similar (*P* = 0.67 and *P* = 0.18, resp.).

Extrapolation from the rat PET data suggested estimated human effective doses for a 70 kg man of 0.024 ± 0.0041 mSv/MBq for ^68^Ga-DOTA-Siglec-9, and 0.022 ± 0.0042 mSv/MBq for ^18^F-FDR-Siglec-9. The most critical organs were the urinary bladder wall with ^68^Ga-DOTA-Siglec-9 (0.20 ± 0.087 mSv/MBq) and kidneys with ^18^F-FDR-Siglec-9 (0.29 ± 0.13 mSv/MBq) (Tables 3 and 4).

### 3.3. Mouse Studies

**Figure 3:**
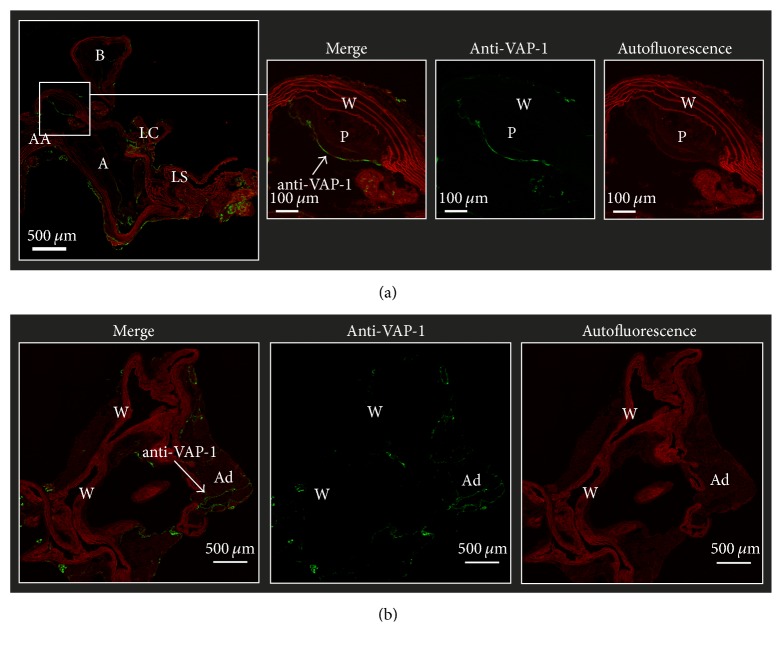
VAP-1 immunofluorescence of (a) atherosclerotic LDLR^−/−^ApoB^100/100^ and (b) C57BL/6N control mice aortas. AA: ascending aorta, A: aortic arch, LC: left common carotid artery, LS: left subclavian artery, P: plaque, W: wall, and Ad: adipocyte.

The LDLR^−/−^ApoB^100/100^ mice demonstrated atherosclerotic plaques, especially in the aortic arch, while the C57BL/6N mice showed no evidence of atherosclerosis. Furthermore, the LDLR^−/−^ApoB^100/100^ atherosclerotic lesions were VAP-1 positive, whereas normal vessel walls in the aortas of C57BL/6N mice were VAP-1-negative (Figure 3).

**Figure 4:**
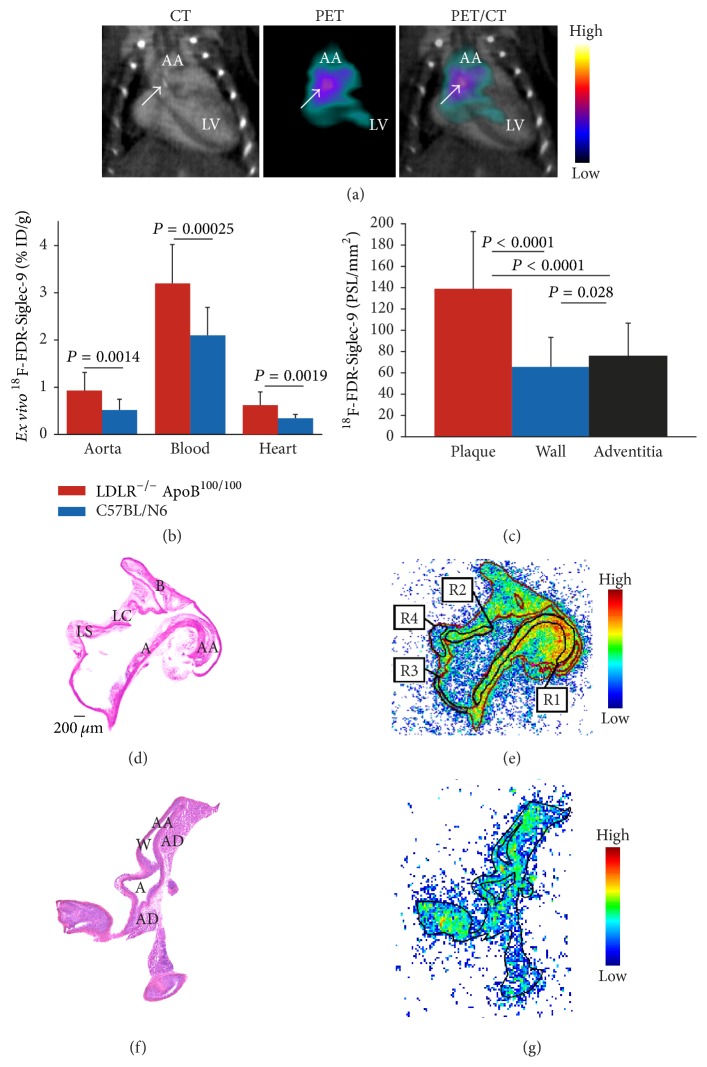
(a) The ^18^F-FDR-Siglec-9 PET/CT detects atherosclerotic plaques in the aortic root with a target-to-background ratio (SUV_max,aortic  arch_/SUV_mean,blood_) of 1.6. The blood radioactivity concentration was determined from heart left ventricle using contrast-enhanced CT as anatomical reference. Arrow shows calcified atherosclerotic plaques in the aortic arch (a). (b)* Ex vivo* biodistribution of ^18^F-FDR-Siglec-9 in LDLR^−/−^ApoB^100/100^  (*n* = 19) and C57BL/N6 (*n* = 13) mice. (c) Quantification of ^18^F-FDR-Siglec-9 binding on the autoradiography of atherosclerotic mice aortas (*n* = 12,* P* values one-way ANOVA with Tukey's correction). (d) Representative hematoxylin-eosin staining of a longitudinally sectioned LDLR^−/−^ApoB^100/100^ mouse aorta and (e) a superimposed autoradiograph (red lines represent the borders of the hematoxylin-eosin image). R1 and R2 are regions of interest in the plaque (excluding the media), R3 is in normal vessel wall (no lesion formation), and R4 is in adventitia (mainly adipose tissue around the aorta). (f) Hematoxylin-eosin staining of a longitudinally sectioned healthy C57BL/6N control mouse aorta. (g) Superimposed autoradiograph and hematoxylin-eosin staining (the black line represents the borders of the hematoxylin-eosin image). A: arch; AA: ascending aorta; B: brachiocephalic artery; LC: left common carotid artery; and LS: left subclavian artery. PSL/mm^2^ = photostimulated luminescence/mm^2^ normalized for injected radioactivity dose.

^18^F-FDR-Siglec-9 PET/CT imaging of atherosclerotic mice showed plaques in the aortic arch, with a target-to-background ratio of 1.6 ± 0.078 at 10–20 min after injection (Figure 4(a)). This ratio was similar to that reported previously for ^68^Ga-DOTA-Siglec-9 (1.7 ± 0.22, *P* = 0.35).

According to* ex vivo* gamma counting, the aortic uptake of ^18^F-FDR-Siglec-9 was significantly higher (*P* = 0.0015) in the LDLR^−/−^ApoB^100/100^ mice (0.93 ± 0.38  % ID/g) than in the C57BL/6N mice (0.52 ± 0.23  % ID/g; Table 5) and comparable to the uptake of ^68^Ga-DOTA-Siglec-9 (0.83 ± 0.33  % ID/g, *P* = 0.38).

Autoradiography of aortic cryosections further confirmed ^18^F-FDR-Siglec-9 accumulation in atherosclerotic plaques, with a plaque-to-healthy vessel wall ratio of 1.9 ± 0.23 (*P* < 0.001) and plaque-to-adventitia ratio of 2.2 ± 0.53 (*P* < 0.001). In control mice, the uptake of ^18^F-FDR-Siglec-9 in healthy vessel wall was similar to that in the atherosclerotic mice (Figure 4). However, the plaque-to-healthy vessel wall ratios were higher with ^68^Ga-DOTA-Siglec-9 (2.1 ± 0.43) than with ^18^F-FDR-Siglec-9 (*P* = 0.038).

## 4. Discussion

VAP-1 targeted ligands are promising tools for PET imaging of inflammation. In this study, we compared the VAP-1 targeting tracers ^68^Ga-DOTA-Siglec-9 and ^18^F-FDR-Siglec-9 in the detection of experimental inflammation in rats and mice and also estimated the radiation burden to humans. We found that the uptake of both tracers was higher in skin/muscle inflammation than in healthy muscle, and in atherosclerotic rather than in nonatherosclerotic arterial walls. Both tracers resulted in a low radiation exposure, but the lower-cost and more straightforward radiolabeling procedures support the potential use of ^68^Ga-DOTA-Siglec-9 for PET imaging of patients with inflammation.

The tested tracers have a similar amino acid sequence but a differently conjugated peptide structure (^68^Ga-DOTA versus ^18^F-FDR). We hypothesized that the ^18^F-labeled tracer would provide improved visualization of inflammatory foci because it has a lower positron range (0.27 mm) than ^68^Ga (1.05 mm). For PET imaging, ^18^F (*t*_1/2_ = 110 min, *β*^+^_max_ = 640 keV, *β*^+^ = 97%) is an ideal radionuclide, providing a high spatial resolution in the resulting images. ^68^Ga (*t*_1/2_ = 68 min, *β*^+^_max_ = 1899 keV, *β*^+^ = 89%) is a positron-emitting radiometal that is particularly suitable for the labeling of chelate-conjugated peptides. While production of ^18^F requires a cyclotron, ^68^Ga is produced with an easily accessible low-cost ^68^Ge/^68^Ga-generator [17].

Although the inflammation detection characteristics of ^68^Ga-DOTA-Siglec-9 and ^18^F-FDR-Siglec-9 were similar, the uptake of ^18^F-FDR-Siglec-9 was higher in several nontarget tissues, including the control area. We do not have a clear explanation for the distinctive distribution patterns, particularly in the liver, pancreas, heart, and kidneys, but suspect that they were at least partly due to the sugar moiety. Similar results have been observed with ^68^Ga-DOTANOC and ^18^F-FDR-NOC [18]. ^68^Ga-DOTA-Siglec-9 and ^18^F-FDR-Siglec-9 showed comparable* in vivo* imaging of inflammation in the rat model. The difference in the control area between the two tracers might at least partly be explained by the higher blood pool radioactivity of ^18^F-FDR-Siglec-9. Although both ^68^Ga-DOTA-Siglec-9 and ^18^F-FDR-Siglec-9 clearly delineated inflamed area by* in vivo* PET, the ^18^F-FDG uptake was higher (SUV_mean_  2.0 ± 0.52 at 90 min after injection) as reported in our previous rat studies with turpentine-induced inflammation [16].

As we earlier reported, the LDLR^−/−^ApoB^100/100^ mice expressed VAP-1 on endothelial cells lining the inflamed atherosclerotic lesions, while normal vessel walls in the aortas of C57BL/6N mice were VAP-1-negative [4]. In atherosclerotic mice, the aortic uptake of ^18^F-FDR-Siglec-9 was comparable to the previously reported uptake of ^68^Ga-DOTA-Siglec-9 [4]. With both tracers, the atherosclerotic lesions in mice were best detectable by* in vivo* PET/CT imaging at 10–20 min after injection. Autoradiography revealed that the plaque-to-healthy vessel wall ratios were slightly higher with ^68^Ga-DOTA-Siglec-9 than with ^18^F-FDR-Siglec-9, although both were close to the previously reported ratio for ^18^F-FDG (2.3 ± 0.5) in a LDLR^−/−^ApoB^100/100^ model [13].

In general,* in vivo* PET imaging of such a small target as atherosclerotic lesion in mice aorta is very challenging. When size of the imaged structures is smaller than the spatial resolution of the scanner, spillover from adjacent tissues and partial volume effect may invalidate the quantification of PET data in addition to cardiac and respiratory movement artifacts. Although small-animal PET/CT image of atherosclerotic mouse showed hot spot in the lesion-rich aortic arch (Figure 4(a)), the* ex vivo* biodistribution showed that uptake in the whole thoracic aorta was much lower than the blood level (Figure 4(b)). Therefore, it is possible that the PET/CT imaging of atherosclerotic lesions is interfered with blood pool radioactivity. On the contrary, in the rat model, the size and location of focal skin/muscle inflammation as well as the blood radioactivity concentration were much more favorable for reliable PET imaging of inflamed area. The PET scanning protocols and quantification methods used in this study were based on our previous research to allow direct comparison of new and already existing results.

Extrapolated from rat PET data, the human radiation dose estimates for both ^68^Ga-DOTA-Siglec-9 (0.024 ± 0.0041 mSv/MBq) and ^18^F-FDR-Siglec-9 (0.022 ± 0.0042 mSv/MBq) were similar to those for other ^68^Ga-labeled tracers (e.g., ^68^Ga-DOTANOC, 0.025 mSv/MBq) or ^18^F-FDG (0.019 mSv/MBq) [19, 20].

## 5. Conclusion

VAP-1 targeted ^68^Ga-DOTA-Siglec-9 and ^18^F-FDR-Siglec-9 peptides are potential tracers for the PET imaging of inflammation. The human radiation dose estimates indicate a low radiation exposure with either of the investigated tracers. The present study further strengthens the concept of a VAP-1-based imaging strategy for the* in vivo* detection of inflammation by PET.

## Figures and Tables

**Table 1:** Characteristics of study animals.

	Sprague-Dawley skin inflammation rats	LDLR^−/−^ApoB^100/100^ atherosclerotic mice	C57BL/6N control mice
Age (months)	2.2 ± 0.051	5.6 ± 0.96	2.1 ± 0.39
High fat diet (months)	ND	3.6 ± 1.0	ND
Female/male (no.)	0/16	15/4	6/7
Weight (g)	350 ± 22	27 ± 4.0	24 ± 2.2
*In vivo* PET (no.)	13	2	2
*Ex vivo* gamma counting (no.)	16	19	13
*Ex vivo* autoradiography (no.)	ND	12	10

LDLR^−/−^ApoB^100/100^ = low-density lipoprotein receptor-deficient mouse expressing only apolipoprotein B100; ND = not done; no. = number of investigated animals.

**Table 2:** *Ex vivo* biodistribution (% ID/g) of ^68^Ga-DOTA-Siglec-9 and ^18^F-FDR-Siglec-9 at 60 min after injection in rats with skin/muscle inflammation.

Organ	^68^Ga-DOTA-Siglec-9	^18^F-FDR-Siglec-9	*P* value^*∗*^
Control area	0.051 ± 0.0063	0.11 ± 0.056	0.015
Inflamed area	0.12 ± 0.032	0.19 ± 0.053	0.013
Adipose tissue, BAT	0.024 ± 0.026	0.049 ± 0.019	0.12
Adipose tissue, WAT	0.027 ± 0.014	0.017 ± 0.0041	0.090
Blood	0.099 ± 0.040	0.12 ± 0.023	0.33
Bone (femoral)	0.020 ± 0.0089	0.017 ± 0.010	0.67
Bone marrow	0.039 ± 0.012	0.056 ± 0.011	0.022
Brain	0.0062 ± 0.0049	0.010 ± 0.0030	0.083
Heart	0.030 ± 0.011	0.048 ± 0.010	0.0038
Kidneys	2.8 ± 1.8	14 ± 9.9	0.0053
Liver	0.32 ± 0.16	0.68 ± 0.12	0.00014
Lungs	0.079 ± 0.035	0.11 ± 0.028	0.059
Muscle	0.019 ± 0.0091	0.021 ± 0.0067	0.68
Pancreas	0.024 ± 0.0024	0.048 ± 0.011	0.00062
Plasma	0.13 ± 0.015	0.23 ± 0.087	0.087
Small intestine	0.060 ± 0.016	0.091 ± 0.029	0.051
Spleen	0.19 ± 0.110	0.060 ± 0.012	0.0048
Testis	0.035 ± 0.031	0.046 ± 0.022	0.45
Urine	24 ± 11	23 ± 14	0.93

% ID/g = percentage of injected radioactivity dose per gram of tissue; BAT=brown adipose tissue; WAT = white adipose tissue. ^*∗*^Unpaired, 2-tailed Student's *t*-test.

**Table 3:** Normalized number of disintegrations (hours) in source organs extrapolated from the rat PET data.

Organ	^68^Ga-DOTA-Siglec-9	^18^F-FDR- Siglec-9	*P* value^*∗*^
Heart wall	0.0029 ± 0.00072	0.0026 ± 0.0010	0.55
Kidneys	0.093 ± 0.081	0.47 ± 0.21	0.0032
Liver	0.033 ± 0.0097	0.11 ± 0.043	0.0027
Lungs	0.0026 ± 0.0011	0.0029 ± 0.0011	0.62
Muscle	0.054 ± 0.0088	0.081 ± 0.042	0.17
Urinary bladder wall	0.17 ± 0.076	0.15 ± 0.070	0.70
Total body	1.3 ± 0.085	1.8 ± 0.32	0.0042

^*∗*^Unpaired, 2-tailed Student's *t*-test.

**Table 4:** Human dose equivalent estimates (mSv/MBq) extrapolated from the rat PET data.

Organ	^68^Ga-DOTA-Siglec-9	^18^F-FDR- Siglec-9	*P* value^*∗*^
Adrenals	0.014 ± 0.0012	0.020 ± 0.0029	0.0011
Brain	0.012 ± 0.00077	0.010 ± 0.0018	0.064
Breasts	0.011 ± 0.00064	0.0086 ± 0.0011	0.0036
Gallbladder wall	0.014 ± 0.00065	0.017 ± 0.0014	0.00022
Heart wall	0.0083 ± 0.0010	0.0085 ± 0.00045	0.52
Kidneys	0.15 ± 0.12	0.29 ± 0.13	0.070
Liver	0.012 ± 0.0024	0.021 ± 0.0061	0.014
Lower large intestine wall	0.015 ± 0.00077	0.014 ± 0.00091	0.067
Lungs	0.0044 ± 0.00054	0.0062 ± 0.00016	<0.0001
Muscle	0.0050 ± 0.00018	0.0074 ± 0.00025	<0.0001
Ovaries	0.015 ± 0.00064	0.014 ± 0.00083	0.14
Pancreas	0.014 ± 0.00085	0.018 ± 0.0015	0.00018
Red marrow	0.010 ± 0.00037	0.012 ± 0.00032	<0.0001
Osteogenic cells	0.017 ± 0.00094	0.016 ± 0.0019	0.43
Skin	0.010 ± 0.00054	0.0080 ± 0.00080	0.00019
Small intestine	0.014 ± 0.00047	0.015 ± 0.00049	0.0034
Spleen	0.014 ± 0.0011	0.018 ± 0.0022	0.00074
Stomach wall	0.013 ± 0.00065	0.014 ± 0.00041	0.0057
Testes	0.013 ± 0.00062	0.010 ± 0.0010	0.0012
Thymus	0.011 ± 0.00068	0.010 ± 0.0013	0.014
Thyroid	0.011 ± 0.00072	0.0094 ± 0.0014	0.017
Upper large intestine wall	0.014 ± 0.00046	0.015 ± 0.00045	0.0036
Urinary bladder wall	0.20 ± 0.087	0.081 ± 0.032	0.0037
Uterus	0.018 ± 0.0017	0.017 ± 0.0012	0.31
Total body	0.013 ± 0.00041	0.012 ± 0.00012	0.00073
Effective dose	0.024 ± 0.0041	0.022 ± 0.0042	0.58

^*∗*^Unpaired, 2-tailed Student's *t*-test.

**Table 5:** *Ex vivo* biodistribution (% ID/g) of ^18^F-FDR-Siglec-9 at 25 min after injection in atherosclerotic and control mice.

Organ	LDLR^−/−^ApoB^100/100^ (*n* = 19)	C57BL/6N (*n* = 13)	*P* value^*∗*^
Aorta	0.93 ± 0.38	0.52 ± 0.23	0.0014
Adipose tissue, BAT	0.57 ± 1.4	0.45 ± 0.13	0.16
Adipose tissue, WAT	0.72 ± 0.66	0.63 ± 0.37	0.67
Blood	3.2 ± 0.82	2.1 ± 0.59	0.00025
Brain	0.15 ± 0.060	0.11 ± 0.048	0.026
Heart	0.62 ± 0.28	0.34 ± 0.082	0.0019
Kidney	44 ± 26	43 ± 25	0.86
Liver	3.0 ± 0.97	3.5 ± 2.3	0.37
Lungs	2.4 ± 1.1	1.5 ± 0.45	0.011
Muscle	0.62 ± 0.20	0.43 ± 0.13	0.0056
Pancreas	0.82 ± 0.28	0.57 ± 0.18	0.011
Plasma	5.7 ± 1.4	3.8 ± 0.62	0.00015
Small intestine	1.6 ± 0.49	0.79 ± 0.29	<0.0001
Spleen	1.1 ± 0.28	0.62 ± 0.32	0.054
Thymus	0.69 ± 0.19	0.43 ± 0.089	<0.0001
Urine	470 ± 370	610 ± 340	0.27

% ID/g = percentage of injected radioactivity dose per gram of tissue; BAT = brown adipose tissue; WAT = white adipose tissue. ^*∗*^Unpaired, 2-tailed Student's *t*-test.

## References

[1] Wu C., Li F., Niu G., Chen X. (2013). PET imaging of inflammation biomarkers. *Theranostics*.

[2] Salmi M., Jalkanen S. (2014). Ectoenzymes in leukocyte migration and their therapeutic potential. *Seminars in Immunopathology*.

[3] Anger T., Pohle F. K., Kandler L. (2007). VAP-1, Eotaxin3 and MIG as potential atherosclerotic triggers of severe calcified and stenotic human aortic valves: Effects of statins. *Experimental and Molecular Pathology*.

[4] Silvola J. M. U., Virtanen H., Siitonen R. (2016). Leukocyte trafficking-associated vascular adhesion protein 1 is expressed and functionally active in atherosclerotic plaques. *Scientific Reports*.

[5] Bulgarelli A., Martins Dias A. A., Caramelli B., Maranhão R. C. (2012). Treatment with methotrexate inhibits atherogenesis in cholesterol-fed rabbits. *Journal of Cardiovascular Pharmacology*.

[6] Aalto K., Maksimow M., Juonala M. (2012). Soluble vascular adhesion protein-1 correlates with cardiovascular risk factors and early atherosclerotic manifestations. *Arteriosclerosis, Thrombosis, and Vascular Biology*.

[7] Li H.-Y., Lin M.-S., Wei J.-N. (2009). Change of serum vascular adhesion protein-1 after glucose loading correlates to carotid intima-medial thickness in non-diabetic subjects. *Clinica Chimica Acta*.

[8] Aalto K., Autio A., Kiss E. A. (2011). Siglec-9 is a novel leukocyte ligand for vascular adhesion protein-1 and can be used in PET imaging of inflammation and cancer. *Blood*.

[9] Li X.-G., Autio A., Ahtinen H. (2013). Translating the concept of peptide labeling with 5-deoxy-5-[ ^18^F]fluororibose into preclinical practice: ^18^F-labeling of Siglec-9 peptide for PET imaging of inflammation. *Chemical Communications*.

[10] Ahtinen H., Kulkova J., Lindholm L. (2014). ^68^Ga-DOTA-Siglec-9 PET/CT imaging of peri-implant tissue responses and staphylococcal infections. *EJNMMI Research*.

[11] Virtanen H., Autio A., Siitonen R. (2015). ^68^Ga-DOTA-Siglec-9 - a new imaging tool to detect synovitis. *Arthritis Research & Therapy*.

[12] Yamada S., Kubota K., Kubota R., Ido T., Tamahashi N. (1995). High accumulation of fluorine-18-fluorodeoxyglucose in turpentine-induced inflammatory tissue. *Journal of Nuclear Medicine*.

[13] Silvola J. M. U., Saraste A., Laitinen I. (2011). Effects of age, diet, and type 2 diabetes on the development and FDG uptake of atherosclerotic plaques. *JACC: Cardiovascular Imaging*.

[14] Mikkola K., Yim C.-B., Fagerholm V. (2014). ^64^Cu- and ^68^Ga-labelled [Nle^14^,Lys^40^(Ahx-NODAGA)NH_2_]-exendin-4 for pancreatic beta cell imaging in rats. *Molecular Imaging and Biology*.

[15] Merinen M., Irjala H., Salmi M., Jaakkola I., Hänninen A., Jalkanen S. (2005). Vascular adhesion protein-1 is involved in both acute and chronic inflammation in the mouse. *The American Journal of Pathology*.

[16] Autio A., Ujula T., Luoto P., Salomäki S., Jalkanen S., Roivainen A. (2010). PET imaging of inflammation and adenocarcinoma xenografts using vascular adhesion protein 1 targeting peptide ^68^Ga-DOTAVAP-P1: Comparison with ^18^F-FDG. *European Journal of Nuclear Medicine and Molecular Imaging*.

[17] Sanchez-Crespo A. (2013). Comparison of Gallium-68 and Fluorine-18 imaging characteristics in positron emission tomography. *Applied Radiation and Isotopes*.

[18] Rinne P., Hellberg S., Kiugel M. (2016). Comparison of somatostatin receptor 2-targeting pet tracers in the detection of mouse atherosclerotic plaques. *Molecular Imaging and Biology*.

[19] Pettinato C., Sarnelli A., Di Donna M. (2008). ^68^Ga-DOTANOC: Biodistribution and dosimetry in patients affected by neuroendocrine tumors. *European Journal of Nuclear Medicine and Molecular Imaging*.

[20] International Commission on Radiological Protection Publication 80. “Recalculated dose data for 19 frequently used radiopharmaceuticals from ICRP publication 53”.* Annals of the ICRP* ICRP. vol. 28 pp. 47–83 1998

